# Independent effects on cellular and humoral immune responses underlie genotype-by-genotype interactions between *Drosophila* and parasitoids

**DOI:** 10.1371/journal.ppat.1008084

**Published:** 2019-10-07

**Authors:** Alexandre B. Leitão, Xueni Bian, Jonathan P. Day, Simone Pitton, Eşref Demir, Francis M. Jiggins

**Affiliations:** 1 Department of Genetics, University of Cambridge, Cambridge, United Kingdom; 2 Antalya Bilim University, Faculty of Engineering, Department of Material Science and Nanotechnology Engineering, Dosemealti, Antalya, Turkey; Institut Pasteur, FRANCE

## Abstract

It is common to find abundant genetic variation in host resistance and parasite infectivity within populations, with the outcome of infection frequently depending on genotype-specific interactions. Underlying these effects are complex immune defenses that are under the control of both host and parasite genes. We have found extensive variation in *Drosophila melanogaster*’s immune response against the parasitoid wasp *Leptopilina boulardi*. Some aspects of the immune response, such as phenoloxidase activity, are predominantly affected by the host genotype. Some, such as upregulation of the complement-like protein Tep1, are controlled by the parasite genotype. Others, like the differentiation of immune cells called lamellocytes, depend on the specific combination of host and parasite genotypes. These observations illustrate how the outcome of infection depends on independent genetic effects on different aspects of host immunity. As parasite-killing results from the concerted action of different components of the immune response, these observations provide a physiological mechanism to generate phenomena like epistasis and genotype-interactions that underlie models of coevolution.

## Author summary

In many species individuals differ greatly in how resistant they are to infection. Resistance frequently depends on the combination of host and parasite genomes—a host which is resistant to one parasite genotype may be susceptible to a different genotype. This has important evolutionary consequences, maintaining genetic variation in populations and driving dynamic changes in the frequency of the genetic variants involved. To understand how differences in the immune response give rise to these genetic interactions, we have studied a parasitic wasp that lays its eggs within the larvae of the fruit fly *Drosophila melanogaster*. We found that some aspects of the immune response are affected by the host genome, some by the parasite genome, and some by the specific combination of host and parasite. However, no single component of the immune response could predict whether a host killed the parasite. Our results demonstrate how a complex interplay between host and parasite genomes controls different aspects of immunity, ultimately determining whether the host can resist infection.

## Introduction

When a host is exposed to a parasite, the likelihood of infection and the subsequent severity of disease are frequently affected by both the host and parasite genotypes. Frequently, the outcome of infection depends on the combination of host and parasite genotypes, such that the susceptibility of a host to one parasite genotype does not predict its susceptibility to a different parasite genotype [1]. These genotype by genotype (GxG) interactions, where the outcome of infection is the product of host and parasite genotypes, have important consequences. They underpin many theoretical models of coevolution, and can maintain genetic variation within populations [2] or favour the evolution of recombination and sexual reproduction [3]. Similarly, key traits in epidemiology, such as transmission, virulence and recovery, may frequently be the product of GxG interactions [1].

The success of an infection is frequently determined by the host immune response. These are complex traits that are typically comprised of cellular processes, such as phagocytosis, and humoral processes, such as production of antimicrobial peptides [4]. These immune responses are not only under the control of host genes, as parasites have evolved a myriad of ways to suppress these immune defences or avoid triggering them in the first place [5]. Other aspects of host and parasite biology, from behaviour to anatomy, also affect infection. Therefore, genetic variation affecting the outcomes of infection, such as infectivity, pathogen load or disease severity, is likely the result of multiple independent effects on different aspects of the immune response or other traits.

How these processes generate the specificity of attack and defense seen in GxG interactions is well-characterized in many plant-pathogen systems [6], but is only beginning to be understood in animals. The genetic loci or the precise genetic variants underlying specific resistance have been identified in model systems such as sigma virus resistance in *Drosophila* [7–9] and *Pasteuria ramosa* resistance in the water flea *Daphnia magna* [10]. In *D*. *magna* there are strong GxG interactions mediated by the ability of parasite spores to attach to the host esophagus [11]. Elsewhere, genetic specificity arises from the immune system. Perhaps the best understood case is the vertebrate MHC complex, where different host MHC-I and MHC-II alleles encode molecules that present different repertoires of pathogen-derived peptides to immune cells. During the course of an infection HIV can escape from specific MHC-I molecules by altering the sequence of these peptides, thereby generating GXG interactions [12]. In insects, transcriptomic analyses between bumblebees and their trypanosomatid parasites have suggested that differences in immune response can also give rise to GxG interactions [13]. However, the innate immune system of insects relies to a large extent on conserved pathogen molecules known as PAMPs (pathogen associated molecular patterns) to detect infection [4]. This may make recognition a less common source of GxG interactions than for vertebrate MHC. Instead, the genotype-specific immune response of bumblebees may result to arise from interactions between pathogen molecules that suppress immunity and their targets in the host [13].

Understanding the effects of host and parasite genotype on different immune defences is important because it may alter their evolutionary dynamics. Immune traits controlled by strong GxG interactions are likely to experience fluctuating selection through time and space, while traits under the control of just the host or parasite genomes may be under directional or stabilizing selection [14–16]. Traits with little genetic variation may play little role in coevolution. Of particular interest is whether the genetic variation in the host and parasite population affects the same or different traits, and how effects on different traits combine to determine the outcome of infection and generate GxG interactions. These ideas have been discussed in detail for linear stepwise processes during infection, especially in *Daphnia magna* (eg exposure->attachment->penetration->infection) [14–18]. However, parasite killing frequently relies on the concerted action of multiple immune factors that are not connected in a linear fashion, so how genetic variation in different immune traits alters the outcome of infection may be complex.

We have investigated how host genotype, parasite genotype and G x G interactions affect different components of *Drosophila melanogaster*’s immune response to the parasitoid wasp *Leptopilina boulardi*. Parasitoids are the most important parasites known in natural populations of *D*. *melanogaster*, sometimes infecting the majority of individuals in the population [19]. There is considerable genetic variation in the susceptibility of *D*. *melanogaster* to *L*. *boulardi—Drosophila* populations can rapidly evolve resistance in response to artificial selection [20,21] and there are marked differences in the resistance of different populations [22]. Similarly, the virulence of *L*. *boulardi* varies genetically, again with extensive geographical variation [23]. Whether the host or parasitoid survives depends on the combination of host and parasitoid genotype, with the most virulent parasitoids surviving the immune response even in genetically resistant hosts [24]. This previous work on the interaction of host and parasitoid genotypes has focused on the final outcome of infection—whether the host immune response can kill the parasite. Here, we have sampled host and parasite genotypes across the full range of resistance and virulence to investigate the immunological basis of this variation.

*D*. *melanogaster* larvae have a dedicated immune response to fight parasitoid infection. Upon infection hemocytes (blood cells) proliferate, migrate into circulation, and differentiate into a specialized cell type called lamellocytes [25]. Alongside these cellular changes, humoral factors are upregulated and a proteolytic cascade activates the phenoloxidase enzyme, catalyzing the production of melanin [26]. If the immune response is successful, parasitoid wasp eggs or larvae are surrounded by layers of plasmatocytes and lamellocytes and melanized. To sabotage these immune defenses, *L*. *boulardi* injects fly larvae with venom that suppresses the differentiation of lamellocytes [27], induces changes in lamellocyte morphology [28] and inhibits the phenoloxidase cascade [29]. Here we investigate how these different aspects of the anti-parasitoid immune response are influenced by the host and parasite genotypes.

## Results

### *S*uppression of the encapsulation response explains differences in parasitoid virulence but not host resistance

**Fig 1:**
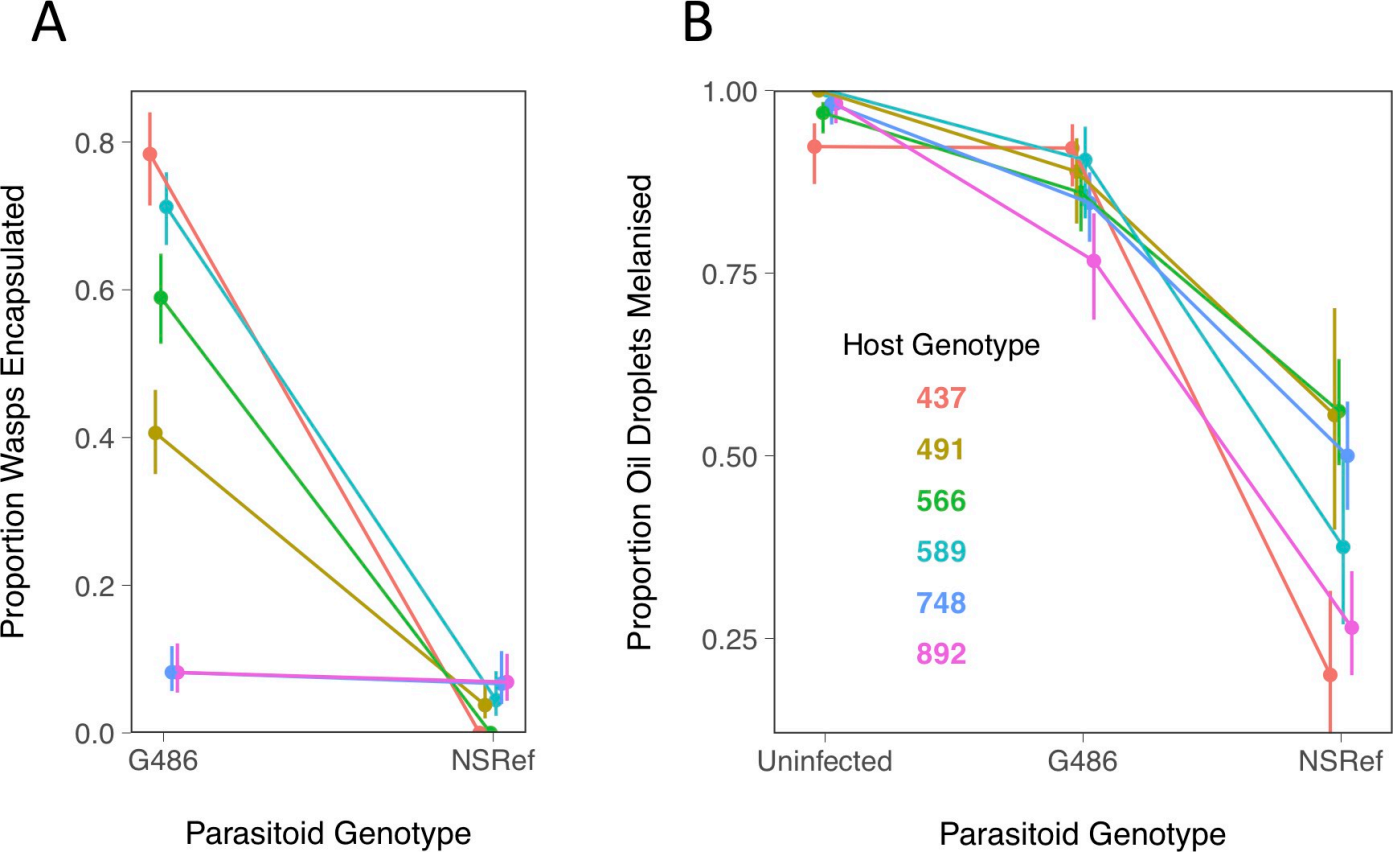
Resistance of six *Drosophila* genotypes to two parasitoid genotypes. (A) Proportion of *Drosophila* larvae encapsulating *L*. *boulardi*. (B) Proportion of oil droplets injected onto *Drosophila* larvae that were melanised. The larvae were previously parasitized by *L*. *boulardi* or were parasitoid-free controls. Bars are standard errors. Samples sizes per data point in panel A were a mean of 55 larvae and in panel B 38 larvae (full details are in S1 Table).

When an insect is infected with a parasitoid, either the host or the parasite will die. To study the effects of the host and parasitoid genotypes on the outcome of infection, we infected six inbred *D*. *melanogaster* lines with two *L*. *boulardi* lines. The parasitoid lines were selected because they differ in virulence [20,30]. The *Drosophila* lines were selected from the *Drosophila* genetic reference panel (DGRP) [31] on the basis of preliminary experiments showing they varied in their resistance. We scored whether *Drosophila* had mounted a successful immune response against the parasitoid by the presence of a black melanized capsule around the wasp egg or larva. When a *Drosophila* larva is parasitised, it is common to find that both the host and parasitoid dies. If this is the case, *Drosophila* often dies as a pupa. Hence, we not only estimated encapsulation rates by dissecting *Drosophila* larvae 48h hours post parasitism (Fig 1A), but we also recorded the proportion of parasitised larvae that survived to adulthood (S1A Fig).

There was a large effect of both the parasitoid and host genotype on encapsulation rates. Confirming published results, the G486 parasitoid line was more frequently encapsulated [30] than the NSRef line [20] (Figs 1A and S1A). The host genotypes also differed considerably in their resistance, with encapsulation rates after G486 infection ranging from 8–78% (Fig 1A). Despite NSRef killing the large majority of flies regardless of the *Drosophila* genotype, there was still a significant GxG interaction affecting the probability of encapsulation (Fig 1A; host genotype x parasitoid treatment interaction: χ^2^ = 47.81, d.f. = 5, *p* = 4x10^-8^). This can be seen as the two genotypes with the lowest chance of encapsulating G486 parasitoids are the most likely to encapsulate NSRef parasitoids (Fig 1A). The results were similar when we measured the survival of flies to adulthood after parasitism (S1A Fig). A high percentage of *Drosophila* larvae were parasitised (77%-95%), and neither the host nor parasite genotype affected the rate that larvae were attacked (S1B Fig; host genotype: χ^2^ = 8.26, d.f. = 5, *p* = 0.14; host genotype x parasite genotype: χ^2^ = 1.69, d.f. = 5, *p* = 0.89).

As *L*. *boulardi* injects venoms that suppress the encapsulation response [5], we tested whether differences in systemic immune suppression could explain the effects of *Drosophila* or *L*. *boulardi* genotype. We first allowed the wasps to parasitise the *Drosophila* larvae and then injected a droplet of oil (the oil contained homogenized *L*. *boulardi* to elicit a strong melanization response). In the parasitoid-free controls the oil droplet triggers a strong encapsulation response in all the *Drosophila* lines (Fig 1B). This is weakly suppressed after G486 infection and strongly suppressed after NSRef infection (Fig 1B; main effect parasitoid genotype, excluding uninfected controls: χ^2^ = 68.93, d.f. = 1, *p*<10^−10^). This indicates that the effect of parasite genotype on encapsulation rates is due to differences in systemic suppression of the host’s immune response.

In contrast, differences in the resistance of the host genotypes to infection do not reflect differences in immune suppression. The *Drosophila* lines do not vary significantly in their ability to melanise oil droplets (Fig 1B; main effect host genotype, excluding uninfected controls: χ^2^ = 9.89, d.f. = 5, *p* = 0.08). Furthermore, the two lines that are very susceptible to G486 infection do not suffer from unusually strong immune suppression (Fig 1A versus 1B). Overall there was no evidence that the *Drosophila* genotype affected the degree of immune suppression by the parasitoid (Fig 1B; host genotype x parasitoid treatment interaction, excluding uninfected controls: χ^2^ = 6.90, d.f. = 5, *p* = 0.23).

### Genotype by genotype interactions control the proliferation and differentiation of hemocytes

**Fig 2:**
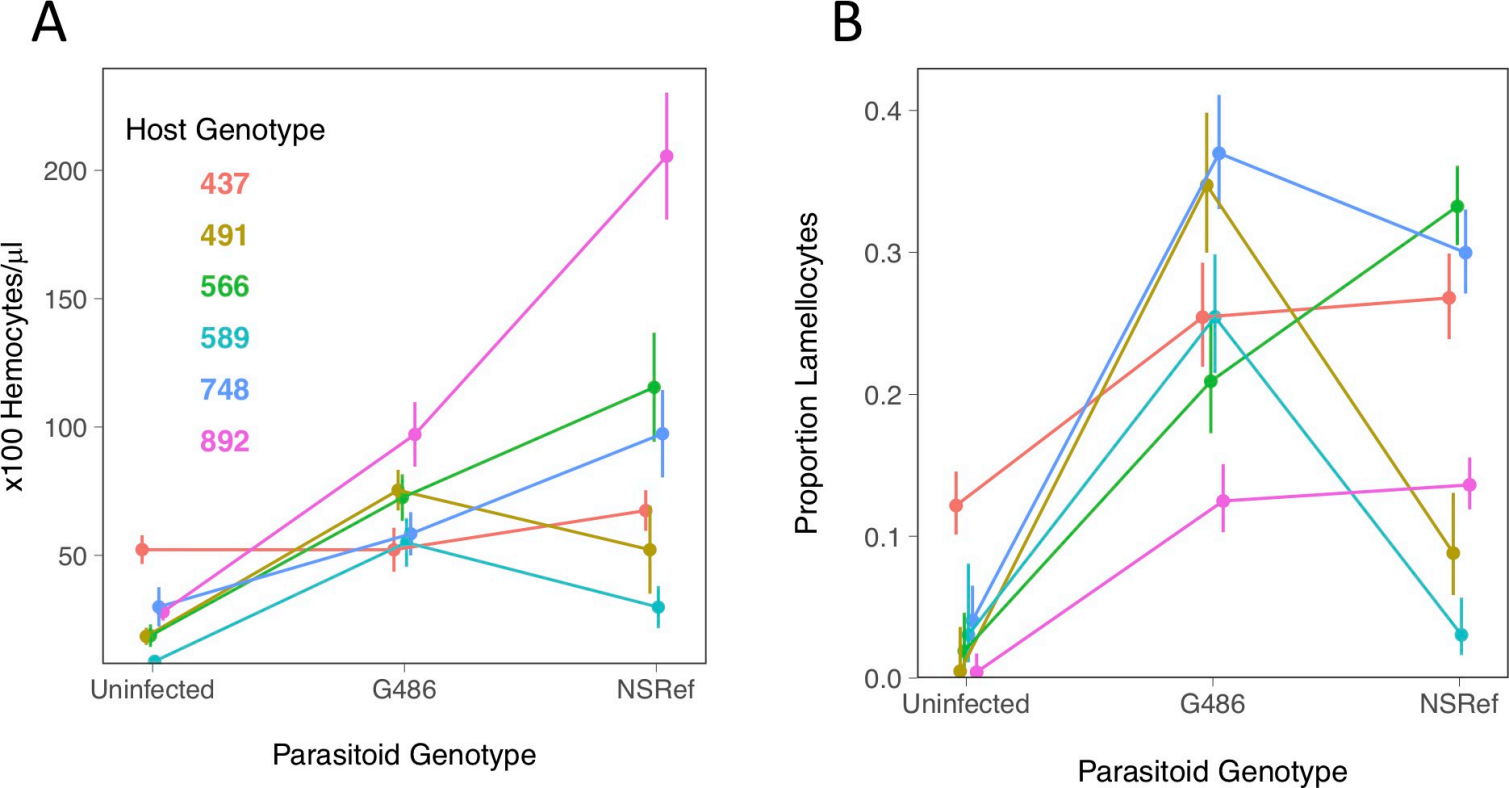
Cellular immune response of six *Drosophila* genotypes to two parasitoid genotypes. (A) The concentration of circulating hemocytes in control and infected *Drosophila* larvae. (B) The proportion of circulating hemocytes that are lamellocytes in control and infected *Drosophila* larvae. The lamellocyte proportions are coefficients estimated from a GLM. Bars are standard errors. There are a mean of 10.7 hemocyte counts per data point (each involved bleeding 10–12 larvae; full details are in S1 Table).

After parasitoid infection *Drosophila* hemocytes proliferate and move into circulation [25]. This increase in hemocyte numbers was strongly influenced by GxG interactions (Fig 2A; host genotype x parasitoid treatment interaction, excluding uninfected controls: χ^2^ = 19.00, d.f. = 5, *p* = 0.002). Two *Drosophila* lines have higher numbers of circulating hemocytes following G486 infection while the remaining lines had more after NSRef infection. Similar effects are seen when comparing the uninfected controls to the infected larvae—the *Drosophila* line with the highest number of hemocytes before infection had relatively low numbers after infection (Fig 2A, line 437). Similar effects are seen when the number of plasmatocytes is measured rather than the total number of hemocytes (S2 Fig). Despite the strong genetic effects on this critical component of the immune response, the changes in hemocyte numbers do not predict the outcome of infection (Figs 1A versus 2A and S3; Pearson Correlation: *r*^*2*^ = 0.09, *p* = 0.35).

Parasitoid infection also causes hemocytes to differentiate into cells with a specialized anti-parasitoid function called lamellocytes. This process was influenced by striking GxG interactions (Fig 2B; host genotype x parasitoid treatment interaction, excluding uninfected controls: χ^2^ = 37.55, d.f. = 5, *p* = 4.7x10^-7^). While infections with both parasitoid genotypes results in the differentiation of lamellocytes in all the *Drosophila* lines (Fig 2B), the magnitude of this effect differs greatly between host-parasitoid combinations. In one *Drosophila* line lamellocytes were abundant without infection, suggesting that this aspect of the immune response was constitutively active (Fig 2B, line 437). If the absolute number of lamellocytes is examined rather than the proportion, the overall pattern is similar (S2B Fig).

As was the case for hemocyte numbers, the induction of lamellocytes does not predict the final outcome of infection (Figs 1A versus 2B and S3; Pearson Correlation: *r*^*2*^ = 0.03, *p* = 0.58). For example, of the two host genotypes with very low survival rates after G486 infection, one had the lowest rate of lamellocyte differentiation and the other had the highest rate (Figs 1A and 2B). Similarly, despite the NSRef parasitoid line being highly virulent against all the *Drosophila* lines, in three lines it induces high rates of differentiation.

### Different components of the humoral immune response are affected by host or parasitoid genotype

**Fig 3:**
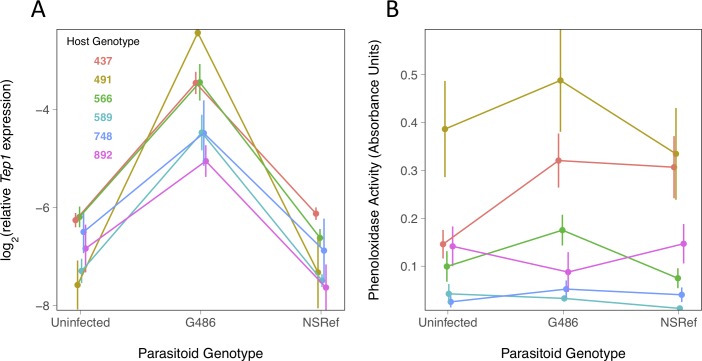
Humoral immune response of six *Drosophila* genotypes to two parasitoid genotypes. (A) *Tep1* expression relative to *RpL32* expression measured by quantitative PCR. (B) Phenoloxidase activity in the hemolymph of *Drosophila* larvae, measured by 490nm light absorbance due to conversion of L-DOPA into dopachrome. Larvae were incubated for 24h post infection in (A) and (B). Bars are standard errors. Each data point in panel A is estimated from 8 pools of 10 larvae and in panel B is from a mean of 10.6 pools of 20–25 larvae (details in S1 Table).

We found only one of our parasitoid genotypes upregulated *Tep1*, which encodes a secreted complement-like protein that is up-regulated upon *L*.*boulardi* infection [32] and is involved in the encapsulation response [33]. The expression of the gene in NSRef-infected larvae did not differ from the uninfected controls (Fig 3A; comparison of NSRef infection to control: *F* = 0.97, d.f. = 1,84, *p* = 0.33). In contrast, following G486 infection *Tep1* expression increased considerably (Fig 3A; comparison of G486 infection to control, main effect wasp infection: *F* = 178.00, d.f. = 1,84, *p*<10^−10^). The magnitude of this induction by G486 varied between 3 and 35 fold depending on the *Drosophila* genotype (Fig 3A; comparison of G486 infection to control, host genotype x wasp infection interaction: *F* = 28.48, d.f. = 5,84, *p* = 0.0004). Inspection of the data shows that these differences are driven by the expression of *Tep1* rather than the reference gene we used to normalize expression (Cambridge Data Repository: https://doi.org/10.17863/CAM.44113). Overall, levels of *Tep1* expression are correlated with the rate parasitoids are encapsulated (S3 Fig; Pearson Correlation: *r*^*2*^ = 0.56, *p* = 0.005). This arises because the gene is only upregulated by the less virulent parasitoid genotype, and there is no association with differences in the resistance of different hosts challenged by the same parasitoid (Figs 3A versus 1A).

The melanization of the parasitoid relies upon activation of the enzyme phenoloxidase. We estimated phenoloxidase activity in hemolymph samples through the conversion of L-DOPA into dopachrome. There is a high variation for the trait between DGRP lines but no effect of the parasite infection (Fig 3B; main effect *Drosophila* genotype: *F* = 30.7, d.f. = 1,182, *p* = 1 x 10^−7^; main effect parasitoid treatment: *F* = 1.63, d.f. = 2,182, *p* = 0.20). Phenoloxidase activity alone does not correlate with the rate that parasitoids are encapsulated (Figs 3B versus 1A and S3; Pearson Correlation: *r*^*2*^ = 0.05, *p* = 0.47).

## Discussion

Parasitoids are an extreme form of parasite where onward transmission requires the host to be killed. In line with previous studies of *D*. *melanogaster* and *L*. *boulardi*, we found that both the host and parasite genotype has a considerable effect on which party survives [26]. Similarly strong genetic affects were also apparent when we examined specific components of the anti-parasitoid immune response. However, the genetic effects on each immune component were largely independent–when one aspect of the immune response was compromised it predicted little about other aspects of immunity. Furthermore, whether the host genotype, the parasite genotype or GxG interactions were important depends on which component of the immune response is measured. GxG interactions may be even more widespread across the immune system than we report as we only sampled a small number of host and parasite genotypes.

Our results suggest that immune suppression is critically important in determining the outcome of infection. Fly larvae that had been parasitised by the most virulent wasp genotype were unable to melanize droplets of oil, indicating that the wasp is systemically suppressing the encapsulation response. This is consistent with previous studies showing that *L*. *boulardi* injects *Drosophila* larvae with venom containing potent immune suppressors, and the composition and activity of venoms produced by different *L*. *boulardi* genotypes varies greatly [34–36]. These immunosuppressive venoms may explain why the virulent wasp genotype in our experiments does not induce the expression of the complement protein Tep1. In the mosquito *Anopheles gambiae* a polymorphism in a Tep strongly affects the melanization response against malaria parasites [37], while in *Drosophila* Tep proteins are important in encapsulating parasitoids [33]. Therefore, the suppression of Tep expression may be the mechanism by which encapsulation is being suppressed in our experiments.

Interactions between immune suppressors and their targets in the host may underlie the strong GxG interactions we observed in other immune traits, especially hemocyte differentiation. Highly specific immune defences have long-puzzled immunologists working on arthropods, as recognition proteins and effectors in the innate immune system are typically effective against broad classes of pathogen [38]. However, molecular arms races between immune suppressors and the host molecules they target provide a mechanism to generate these specificities. This is supported by a study of trypanosomatid resistance in bumblebees, where immune suppression has been proposed to explain GxG interactions [13]. Given the ubiquity of immune suppression and evasion across all groups of parasites [39], this may be a common mechanism giving rise to GxG interactions.

In our data, there was no single immune component that explained differences in host resistance (S3 Fig). While *Tep1* expression is correlated with encapsulation rates, it can only explain the effects of parasite genotype and not host genotype. Other immune parameters are not significantly associated with encapsulation. This is particularly surprising for hemocyte number, as the number of hemocytes strongly increases when populations are artificially selected for parasitoid resistance [20,40]. However, our small sample of genotypes prevents us statistically disentangling how the concerted effect of multiple immune traits determines whether a parasite is killed. The details of how immune traits combine to produce resistance may have important implications for coevolution. For example, if parasite-killing requires both hemocyte differentiation and high phenoloxidase activity, then only genotypes with alleles for both these traits will be resistant. The resulting epistasis may have important consequences for the process of coevolution [41].

In many species GxG interactions between hosts and parasites affect the outcome of infection, and our results illustrate two different physiological mechanisms by which these interactions could arise. First, one component of the immune response could be affected by host genotype and another by parasite genotype, as we saw for Tep1 expression and phenoloxidase activity. If parasite killing requires both aspects of the immune response, the outcome of infection will be determined by a GxG interaction. Second, a single physiological response might only be triggered by specific host-parasite genotype combinations, as we saw for lamellocyte differentiation.

## Material and methods

### Fly and wasp maintenance

Stocks were maintained and experiments performed on cornmeal food (per 1200ml water: 13g agar, 105g dextrose, 105g maize, 23g yeast, 35ml Nipagin 10% w/v). Animals were kept at 25°C, in a 14 hours light/10 hours dark cycle and 70% humidity.

Six inbred lines from the *Drosophila melanogaster* genetic reference panel [31] were used in this study: DGRP-437, DGRP-491, DGRP-566, DGRP-589, DGRP-748, DGRP-892. *Drosophila* stocks were maintained by transferring the flies into fresh food every 15 to 19 days. Two *Leptopilina boulardi* strains were used in this study. The G486 strain [30] has low virulence and NSRef [42] is a highly virulent strain. A susceptible outbred population of *D*. *melanogaster* was used to maintain both wasp strains. Eggs from overnight egg lays were collected from agar plates into 1.5ml microcentrifuge tubes in 500μl PBS. 6μl of eggs in PBS (~60 eggs) were transferred into cornmeal food vials and 2 female wasps and one male wasp were added. Vials were incubated for 25 days at 25°C before adult wasps were collected. Adult wasps were maintained in cornmeal vials with a drop of honey up to 10 days before being used for infections.

### Encapsulation assay

*Drosophila* strains were allowed to lay eggs on agar plates overnight (from 6pm to 9am). Eggs were collected in 1.5ml microcentrifuge tubes in 500μl PBS. 15μl of eggs in PBS (~150 eggs) were transferred to cornmeal food plates (50mm diameter) and kept at 25°C for 48h. At this time point, animals were between 48 hours and 63 hours old. In these conditions, all strains used in this study were either in late first or early second instar stage. Second instar larvae were then selected and gently picked with forceps and transferred into food vials (40 larvae per vial). Three female wasps were added to each vial and removed after three hours. To estimate encapsulation rates in adults, vials were incubated for 12 days and emerging flies squashed between two glass slides to score the presence of a capsule. The encapsulation rate was calculated as the number of flies with capsules divided by the total number of infected larvae in the vial (40 minus the number of flies without capsules). To estimate encapsulation rates directly in larvae, larvae were dissected in PBS (phosphate buffered saline) 48 hours post-infection to determine the presence of encapsulated wasp egg/larva or a live wasp larva.

### Oil droplet melanisation

Wasp extracts were prepared by homogenizing 20 *L*.*boulardi* G486 males in 200μl of paraffin oil (Sigma-Aldrich M5904) with a pestle in a 1.5ml microcentrifuge tube. Extracts were centrifuged for 2m 30s at 500g. The supernatant was transferred into a new 1.5ml microcentrifuge tube and the centrifugation step was repeated. The resulting supernatant was used to backfill a glass needle prepared from borosilicate glass 3.5” capillaries (Drummond Scientific Co. 3-000-203-G/X) pulled in a needle puller (Narishige PC-10). The filled needle was attached to a nanoinjector (Drummond Scientific Co. Nonoject II). Second instar larvae on cornmeal food plates were obtained following the same protocol as for encapsulation assay (see above). To obtain parasitized larvae, 4 female wasps were added to food plates containing larvae for 3 hours before injection. Larvae from non-parasitized and parasitized plates were carefully moved onto filter paper in groups of 10 and injected with 4.6nl of oil containing wasp extract. After injection, ddH_2_O was added to the filter paper and larvae were moved with forceps into cornmeal food vials. Vials were incubated for 48h at 25°C. 3^rd^ instar larvae were removed from food with 15% w/v sugar solution and dissected in PBS droplets to score for the presence of melanized oil droplets and to check that larvae exposed to the parasitoid contained a wasp egg or larva.

### Hemocyte counts

*Drosophila* strains were allowed to lay eggs on agar plates overnight (from 6pm to 9am). Eggs were collected in 1.5ml microcentrifuge tubes in 500μl PBS. 5μl of eggs in PBS (~40 eggs) were transferred into cornmeal food vials and kept at 25°C for 48h. At this time point, animals were between 48 and 63 hours old. In these conditions, all strains used in this study were either in late first instar stage or in early second instar stage. Three female wasps were added to treatment vials. Controls were prepared in the same conditions without infection. 48h post-infection, larvae were removed from food with 15% w/v sugar solution, washed in ultrapure H_2_0 and dried in filter paper. Groups of 10 to 12 larvae were rapidly bled from the ventral side in a porcelain dissection dish; 1μl of hemolymph was removed and diluted in 9μl of neutral red (1.65g/L PBS; Sigma-Aldrich N2889). 10μl of hemolymph dilution was transferred into a Thoma counting chamber and hemocytes were counted in 1mm^2^ area, corresponding to 0.1μl volume. Plasmatocytes and lamellocytes were distinguished by size and shape.

### Phenoloxidase activity

*Drosophila* strains were allowed to lay eggs on agar plates overnight (from 6pm to 9am). Eggs were collected in 1.5ml microcentrifuge tubes in PBS. 5μl of eggs in PBS (~40 eggs) were transferred into cornmeal food vials and kept at 25°C for 72h. At this time point, animals were between 72 and 87 hours old. In these conditions, all strains used in this study were either in late second instar stage or in early third instar stage. The later time point for infection in this experiment was necessary to obtain enough hemolymph from 24 hours post-infection larvae. Treatment vials were infected with 3 female wasps. Controls were prepared in the same conditions without infection. 24 hours post-infection, groups of 20–25 larvae were washed with ultrapure H_2_O, dried in filter paper and bled in a porcelain well dish. ~3.5μl of hemolymph was immediately collected, frozen in liquid nitrogen and kept at -80°C. To assay phenoloxidase activity, 2μl of hemolymph was diluted in 18μl of PBS with protease inhibitor (Roche, complete Tablets, Ref 04693159001) in a 96 well U bottom plate (Falcon, Ref 353077). 80μl of L-DOPA (3,4-Dihydroxy-L-phenylanine, Sigma, Ref D9628, 20mM diluted in phosphate buffer pH 6.6) was added to each sample and absorbance at 492nm recorded for 2 hours in 1min intervals in a plate reader. Measurements between 80 and 90 min were averaged and used as a proxy of phenoloxidase activity. Due to technical constraints, two plate readers were used in this study, a CLARIOstar Plus (BMG LABTECH) and a SpectraMax iD3 (Molecular Devices).

### Tep1 expression

*Drosophila* strains were allowed to lay eggs on agar plates overnight (from 6pm to 9am). Eggs were collected in 1.5ml microcentrifuge tubes in PBS. 5μl of eggs in PBS (~40 eggs) were transferred into cornmeal food vials and kept at 25°C for 48h. At this time point, animals were between 48 and 63 hours old. In these conditions, all strains used in this study were either in late first instar stage or in early second instar stage. 3 female wasps were added to treatment vials for 3 hours. Control vials were prepared in the same conditions without wasps. All vials were incubated at 25°C for 24h. To analyze the expression of *Tep1*, RNA was extracted from pools of 10 larvae collected with 15% w/v sugar solution, cleaned with ddH_2_O and dried in filter paper. Larvae were homogenized in 250μl TRIzol [Ambion 15596018] with ~10 1.0mm zirconia beads [Thistle Scientific] in a tissuelyser [Retsch MM300] and kept at -80°C. For RNA extraction, samples were defrosted and centrifuged for 10min at 4°C at 12,000g. 160μl of supernatant was transferred into 1.5ml microcentrifuge tubes, 62.5μl of chloroform [Fisher Scientific C/4920/08] was added, tubes were shaken for 15s and incubated for 3min. After a 10min centrifugation at 12,000g at 4°C, 66μl of the aqueous phase was transferred into a 1.5μl microcentrifuge tube, 156μl of isopropanol [Honeywell 33539] added and the solution thoroughly mixed. After 10min incubation samples were centrifuged for 10min at 12,000g at 4°C and the supernatant was removed. RNA was washed with 250μl 70% ethanol, centrifuged for 2min at 12.000g at 4°C. Ethanol was removed, samples dried, 20μl of nuclease free water [Ambion AM9930] was added and samples incubated at 45°C for 10min. cDNA was prepared from RNA samples with GoScript reverse transcriptase (Promega) according to manufacture instructions. cDNA was diluted 1:10. Exonic primers for *D*. *melanogaster Tep1* were designed in NCBI Primer-BLAST online tool: (Tep1_qPCR_1_Fw: 5’-ACTGGAAGCCTCATTGGTCG-3’; Tep1_qPCR_1_Rev 5’-ACCGACAATGGGAACAGGAC-3’). The gene *RpL32* was used to normalize gene expression (RpL32_qPCR_F-d: 5’-TGCTAAGCTGTCGCACAAATGG-3’; RpL_qPCR_R-h 5’- TGCGCTTGTTCGATCCGTAAC-3’; [43]. Sensifast Hi-Rox SyBr kit (Bioline) was used to perform the RT-qPCR on a StepOnePlus system (Thermo Fisher). Each sample was duplicated (qPCR technical replica). The PCR cycle was 95°C for 2min followed by 40 cycles of 95°C for 5s, 60°C for 30s. The log_2_ *Tep1* expression estimated with the formula Δ*ct* = -(*ct*_*Tep1*_ –*ct*_*RpL32*_), where *ct* is cycle threshold.

### Statistical analysis

Sample sizes for each experiment are listed in S1 Table. The hemocyte counts, gene expression and PO activity were analyzed with Type II ANOVA. For these measurements, in the figures we show the mean and standard error calculated from the raw data. The proportion data (encapsulation rate, melanisation of oil droplets and proportion of lamellocytes) were analysed by logistic regression, allowing for over-dispersion using quasi-likelihoods and assessing significance using Type II tests. For these measurements, in the figures we show estimate the mean and standard error estimated as model coefficients back-transformed from logits to proportions. In all models host genotype, parasitoid genotype and their interaction were included as explanatory variables. The raw data and scripts to reproduce our figures and analysis are available at the Cambridge Data Repository (https://doi.org/10.17863/CAM.44113)

## Supporting information

S1 Table Sample sizes. (PDF)

S1 Fig Resistance of six *Drosophila* genotypes to two parasitoid genotypes. (A) Proportion of parasitized *Drosophila* adults emerging with a capsule (B) Proportion of parasitized *Drosophila*. Bars are standard errors. Samples sizes are detailed in S1 Table.
(JPG)

S2 Fig Circulating hemocyte concentration of six *Drosophila* genotypes in response to infection with two parasitoid genotypes. (A) The concentration of circulating plasmatocytes and (B) lamellocytes in control and infected *Drosophila* larvae. Bars are standard errors. Samples sizes vary between 5 and 17 hemocyte counts (each replicate involved bleeding 10–12 larvae); detailed in S1 Table.
(JPG)

S3 Fig The correlation between immune traits and the proportion of wasps encapsulated. Pearson correlations are given above plots.
(JPG)
