# Supplementary material for: Independent effects on cellular and humoral immune responses underlie genotype-by-genotype interactions between Drosophila and parasitoids
Source: PLoS Pathog. 2019 Oct 7;15(10):e1008084. doi: 10.1371/journal.ppat.1008084 (PMC6797232; doi:10.1371/journal.ppat.1008084)
Supplement: S1 Table — (PDF) [file ppat.1008084.s001.pdf]

				Host Genotype (DGRP line)					
Phenotype	Figure	Sample Type	Treatment	437	491	566	589	748	892
Larval encapsulation	1A	Single larva dissection	G486	37	64	56	73	73	61
			NSRef	41	53	56	45	45	58
Oil melanization	1B	Single larva dissection	No Infection	39	43	65	46	54	56
			G486	38	27	50	21	52	30
			NSRef	15	9	41	16	40	34
Hemocyte counts	2	Hemolymph pooled from 10-12 larvae	No Infection	17	11	14	15	14	17
			G486	11	5	6	8	10	8
			NSRef	13	5	10	11	10	7
Tep1 expression	3A	RNA pooled from 10 larvae	No Infection	8	8	8	8	8	8
			G486	8	8	8	8	8	8
			NSRef	8	8	8	8	8	8
PO activity	3B	Hemolymph pooled from 20-25 larvae	No Infection	12	10	12	8	12	11
			G486	12	9	12	7	12	10
			NSRef	12	8	12	10	12	10
Adult encapsulation	S1	Groups of 40 animals	G486	8	5	8	8	5	3
			NSRef	8	5	8	8	5	4
