# Supplementary figures and images for: Independent effects on cellular and humoral immune responses underlie genotype-by-genotype interactions between Drosophila and parasitoids
Source: PLoS Pathog. 2019 Oct 7;15(10):e1008084. doi: 10.1371/journal.ppat.1008084 (PMC6797232; doi:10.1371/journal.ppat.1008084)

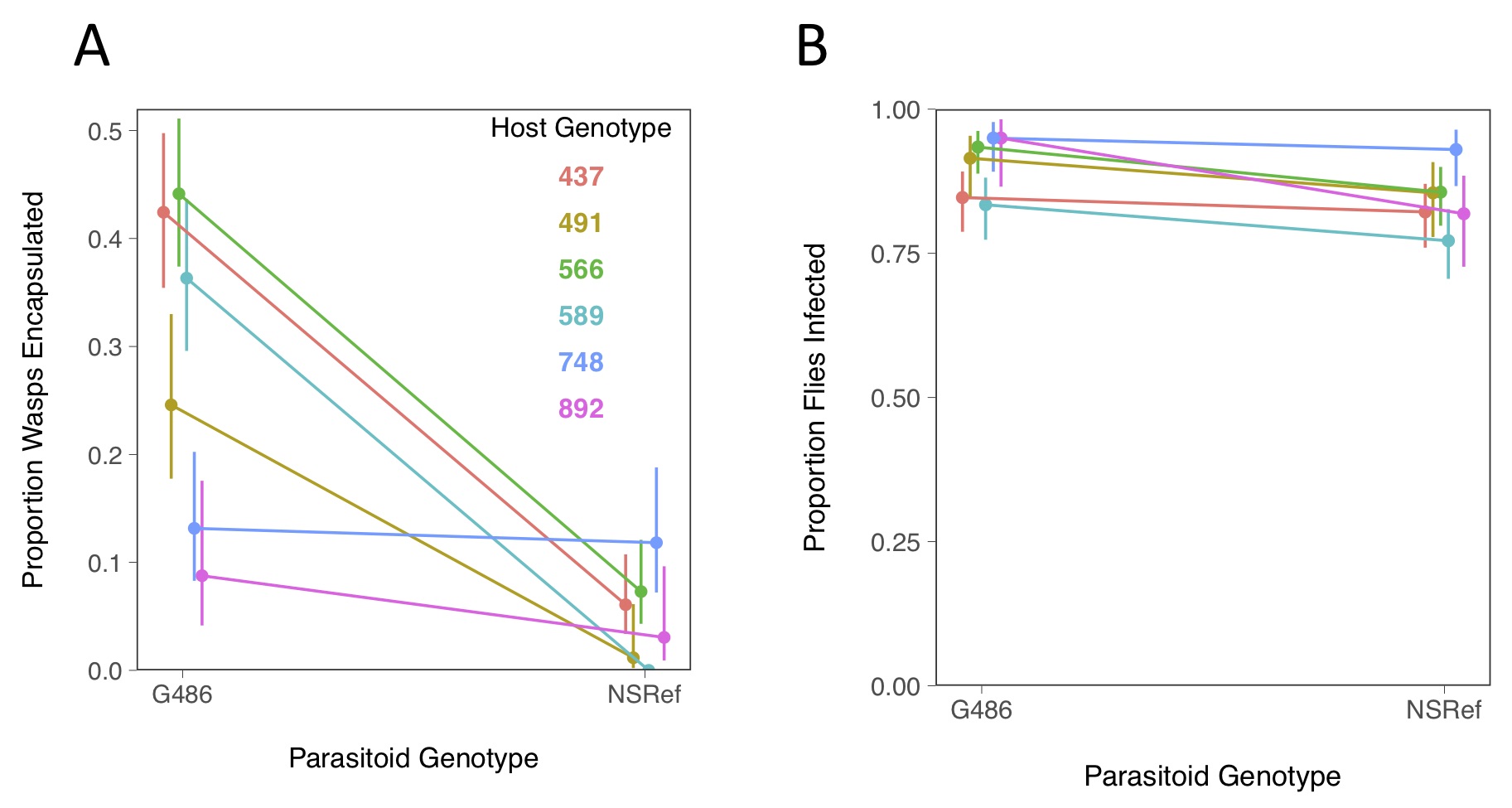

Supplement: S1 Fig — (A) Proportion of parasitized Drosophila adults emerging with a capsule (B) Proportion of parasitized Drosophila. Bars are standard errors. Samples sizes are detailed in S1 Table. (JPG) [file ppat.1008084.s002.jpg]

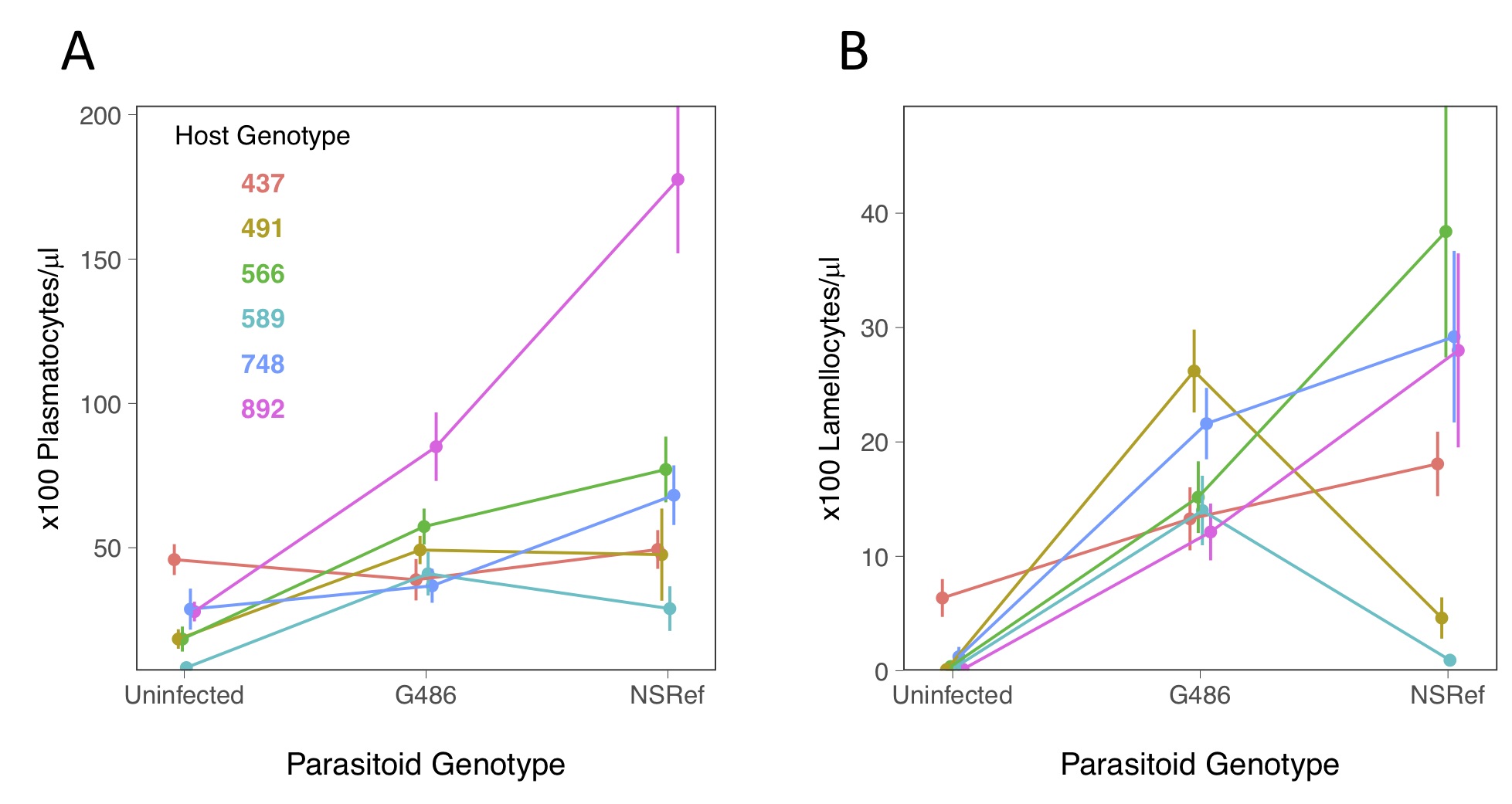

Supplement: S2 Fig — (A) The concentration of circulating plasmatocytes and (B) lamellocytes in control and infected Drosophila larvae. Bars are standard errors. Samples sizes vary between 5 and 17 hemocyte counts (each replicate involved bleeding 10–12 larvae); detailed in S1 Table. (JPG) [file ppat.1008084.s003.jpg]

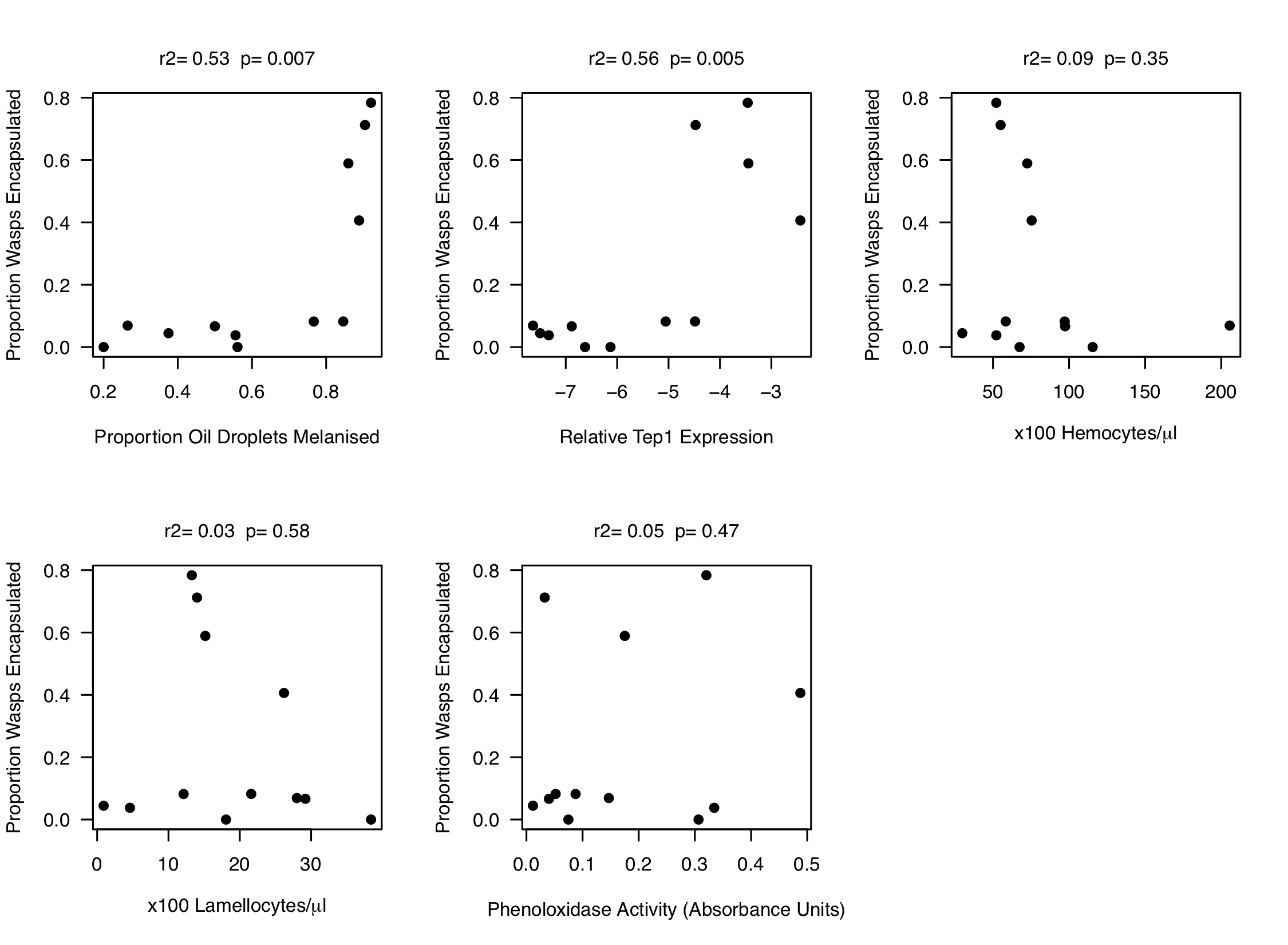

Supplement: S3 Fig — Pearson correlations are given above plots. (JPG) [file ppat.1008084.s004.jpg]
